# The endothelial glycocalyx degenerates with increasing sepsis severity

**DOI:** 10.1186/cc10391

**Published:** 2011-10-27

**Authors:** M Köhler, I Kaufmann, J Briegel, M Jacob, J Goeschl, W Rachinger, M Thiel, M Rehm

**Affiliations:** 1 Clinic of Anesthesiology, University of Munich, Germany; 2 Neurosurgery, University of Munich, Germany; 3 Department of Anesthesiology and Critical Care Medicine, University of Heidelberg, Mannheim, Germany

## Introduction

The endothelial glycocalyx is a recently discovered structure at the luminal side of blood vessels consisting of proteoglycans and glycosaminoglycans, which play an important role in vascular barrier function and cell adhesion. Due to its vulnerability, the endothelial glycocalyx may easily be altered by hypoxia [1], TNFα [2], oxidized lipoproteins [3] and other nonphysiological conditions. We raised the question of whether the glycocalyx may be shed from the endothelium in dependence of severity of sepsis.

## Methods

This clinical prospective study - approved by the local ethics committee - was performed to assess plasma levels of the glycocalyx components (hyaluronane, syndecan, heparan sulfate) by ELISA technique and polymorphonuclear leukocyte (PMN) function by flow cytometry in eight healthy volunteers (HV) and 37 patients who were prospectively enrolled within 24 hours of onset of signs of infection, if they met the criteria for sepsis (*n *= 10), severe sepsis (*n *= 9) and septic shock (*n *= 18) as defined by the members of the ACCP/SCCM Consensus Conference Committee (Table 1). Blood was drawn within 24 hours after onset of sepsis. Informed consent was obtained from all patients or their legal representatives, respectively.

**Table 1:** Demographic data

	Healthy volunteers (*n *= 8)	Sepsis (*n *= 10)	Severe sepsis (*n *= 9)	Septic shock (*n *= 18)
Age (years)	29.1 ± 2.9	51.6 ± 19.7	63.3 ± 23.5	63.3 ± 21.4
APACHE II	n.b.	7.6 ± 3.9	17.8 ± 6.9	27.9 ± 5.3
MOD	n.b.	2.1 ± 1.6	6.9 ± 3.2	9.4 ± 3.6
SOFA	n.b.	4.1 ± 2.8	9.0 ± 3.0	13.3 ± 3.4

## Results

**Figure 1:**
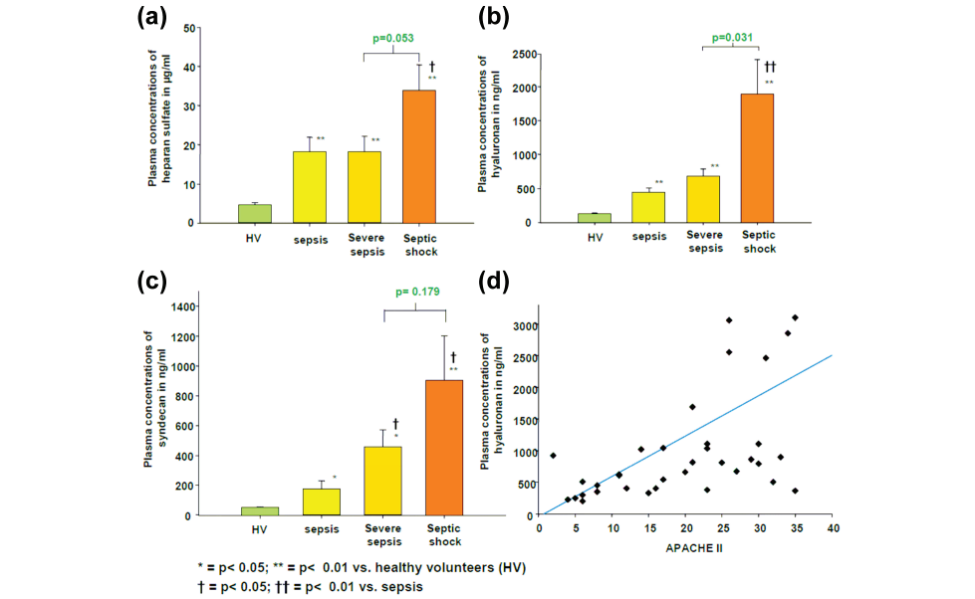
**(a) to (c) Increase in the glycocalyx components in plasma of healthy volunteers (HV) and of patients with increasing sepsis severity**. **(d) **Correlation between APACHE II score of septic patients and hyaluronan plasma concentrations.

Plasma levels of the glycocalyx components were significantly higher in septic patients than in healthy volunteers and even more pronounced in patients with severe sepsis and septic shock (all *P *< 0.05; Figure 1). Hyaluronan and syndecan plasma levels correlated positively with the APACHE II, SOFA and MOD scores (Figure 1 and Table 2). Hyaluronan displayed a positive correlation with the C-reactive protein, procalcitonin and IL-6 in plasma (Table 3). The PMN dysfunction - characterized by an increase in cytotoxic capability and a decrease in microbicidity - showed a parallel course to the heparan sulfate plasma levels.

**Table 2:** Correlation between the glycocalyx components (hyaluronan, syndecan) and the APACHE II, SOFA and MOD score of septic patients

	APACHE II	SOFA	MOD
Hyaluronan	*r*^2 ^= 0.583, *P *= 0.000	*r*^2 ^= 0.529, *P *= 0.001	*r*^2 ^= 0.435, *P *= 0.008
Syndecan	*r*^2 ^= 0.425, *P *= 0.010	*r*^2 ^= 0.476, *P *= 0.003	*r*^2 ^= 0.529, *P *= 0.001

**Table 3:** Correlation between the glycocalyx components (heparan sulfate, hyaluronan) and the C-reactive protein, procalcitonin and IL-6 in plasma of septic patients

	CRP	PCT	IL-6
Heparan sulfate	*r*^2 ^= -0.63, *P *= 0.714	*r*^2 ^= 0.20, *P *= 0.928	*r*^2 ^= 0.505, *P *= 0.012
Hyaluronan	*r*^2 ^= 0.398, *P *= 0.016	*r*^2 ^= 0.723, *P *= 0.000	*r*^2 ^= 0.468, *P *= 0.021

## Conclusion

Elevated plasma levels of hyaluronan, syndecan and heparan sulfate are suggestive of a glycocalyx shedding from endothelium with increasing sepsis severity. This process might contribute to the vascular dysfunction and development of edema in septic patients.
